# Supplementary material for: Revealing the role of double-layer microenvironments in pH-dependent oxygen reduction activity over metal-nitrogen-carbon catalysts
Source: Nat Commun. 2023 Oct 31;14:6936. doi: 10.1038/s41467-023-42749-7 (PMC10618200; doi:10.1038/s41467-023-42749-7)
Supplement: Supplementary file 1 — Supplementary Information [file 41467_2023_42749_MOESM1_ESM.pdf]

Supplementary Information

Revealing the role of double-layer microenvironments in pH-dependent oxygen reduction activity over metal-nitrogen-carbon catalysts

Peng Li[1,‡], Yuzhou Jiao[1,‡], Yaner Ruan[2], Houguo Fei[1], Yana Men[1], Cunlan Guo[1], Yuen Wu[2,*], Shengli Chen[1,*]

[1]Hubei Key Laboratory of Electrochemical Power Sources, College of Chemistry and Molecular Sciences, Wuhan University, Wuhan 430072, China

[2]School of Chemistry and Materials Science, Collaborative Innovation Center of Chemistry for Energy Materials (iChEM), University of Science and Technology of China, Hefei, 230026, China

[‡]These authors contributed equally.

*Corresponding Authors: yuenwu@ustc.edu.cn; slchen@whu.edu.cn

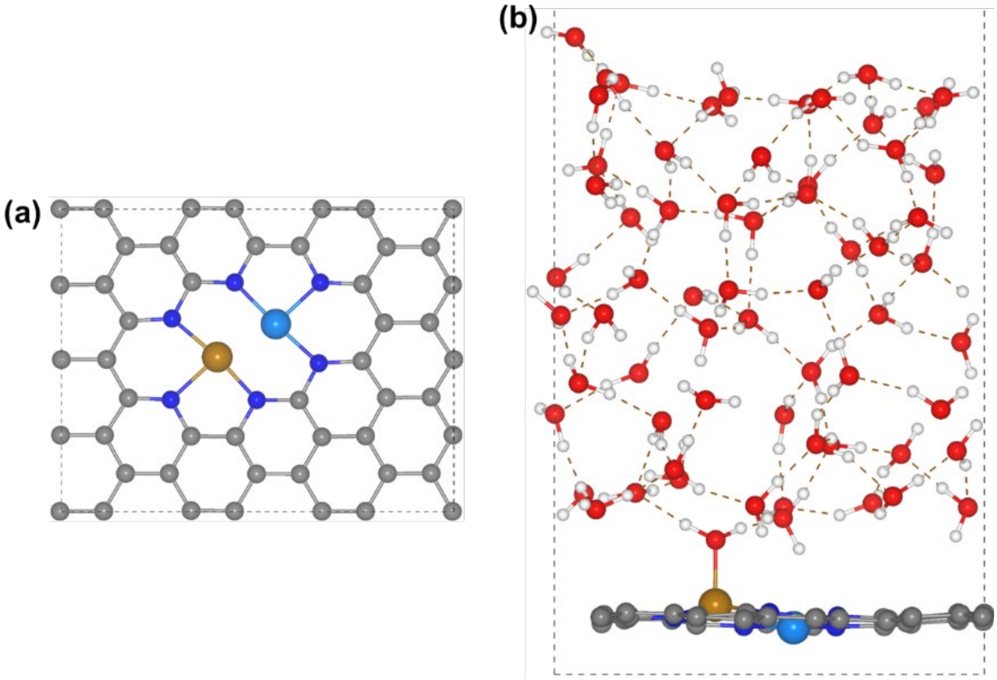

Supplementary Figure 1. (a) Structure model of FeCo-N_6-C catalyst, in which the active site density (SD) is 9.2×10^{20} site/g. (b) Representative snapshot of FeCo-N_6-C/water interface at the potential of zero free charge (PZFC). The Fe, Co, N, C, O and H atoms are colored with brown, sky blue, blue, gray, red and white, respectively (similarly hereinafter). The brown dashed lines represent the hydrogen bonds.

The PZFC of FeCo-N_6-C/water interface is calculated as -0.24 V vs SHE. Unfortunately, to our best knowledge, it seems that there are still no experimentally measured PZFC values for M-N-C catalysts. To evaluate the accuracy of our calculated PZFC values for M-N-C systems, we have also simulated the Fe-N_4-C/water and Co-N_4-C/water interfaces and obtained their PZFCs as contrasts. The representative snapshots of Fe-N_4-C/water and Co-N_4-C/water interfaces are shown in Supplementary Fig. 4a,b, and their PZFCs are calculated as -0.71 V and -0.34 V vs SHE, respectively, which are similar to the values reported by Chan and Liu[1,2]. This confirms that our calculated PZFCs for M-N-C catalysts are accurate and reliable. Therefore, the PZFC of FeCo-N_6-C can be determined as -0.24 V vs SHE based on our AIMD simulations.

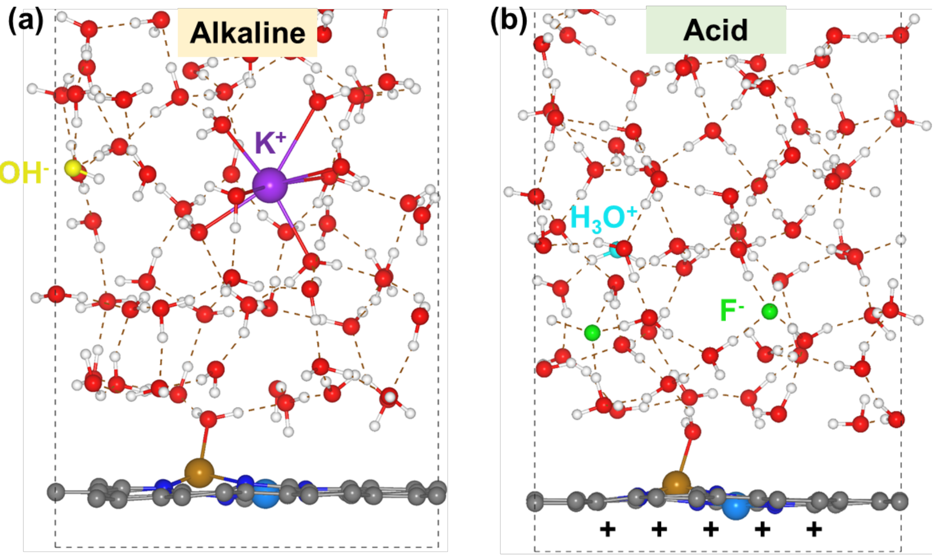

Supplementary Figure 2. Representative snapshots of the interfacial structures at ORR potentials on the bare FeCo-N_6-C electrode surface for (a) alkaline system and (b) acid system. The K^+, OH^-, H_3O^+ and F^- are colored with purple, yellow, green and cyan, respectively (similarly hereinafter). The brown dashed lines represent the hydrogen bonds.

In this work, the definition and adjustment of the electrode potentials corresponding to the experimental ORR conditions are based on the interfaces with $*O_2$ (Fig. 1 in the text), rather than the clean interfaces where no adsorbates exist as shown in Supplementary Fig. 2. This is mainly for two reasons. First, it can be realized that there should always exist oxygen-containing intermediates (e.g., *OOH, *OH, *O) along the whole ORR process. Secondly, due to the electron-withdrawing effect, the interfaces with these oxygen-containing intermediates often possess distinctly higher potentials comparing to that without any oxygen-containing intermediates. For example, the potentials of interfaces shown in Fig. 1b and Supplementary Fig. 2b are 0.88 V and 0.58 V, respectively. It means that under the ORR condition, the interfacial microenvironment will change from the bare system to the oxygen-containing adsorbed systems when the electrode potential is controlled at a same value. In other words, if we define the electrode potential using the interface where no adsorbate exists, the potentials of the subsequent surfaces with adsorbates should also be readjusted again. Therefore, we define the electrode potential using the interface with $*O_2$ intermediate, which is the first reaction intermediate state along ORR process.

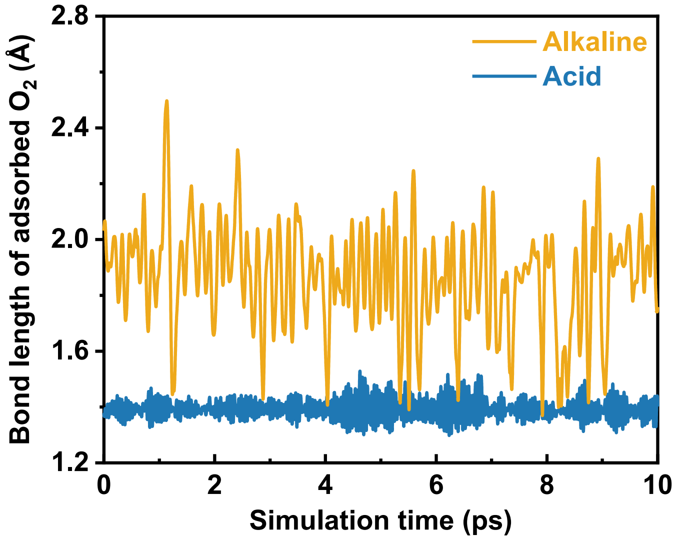

Supplementary Figure 3. Comparison of the bond lengths of adsorbed O_2 at alkaline and acid interfaces among the whole 10 ps AIMD simulations.

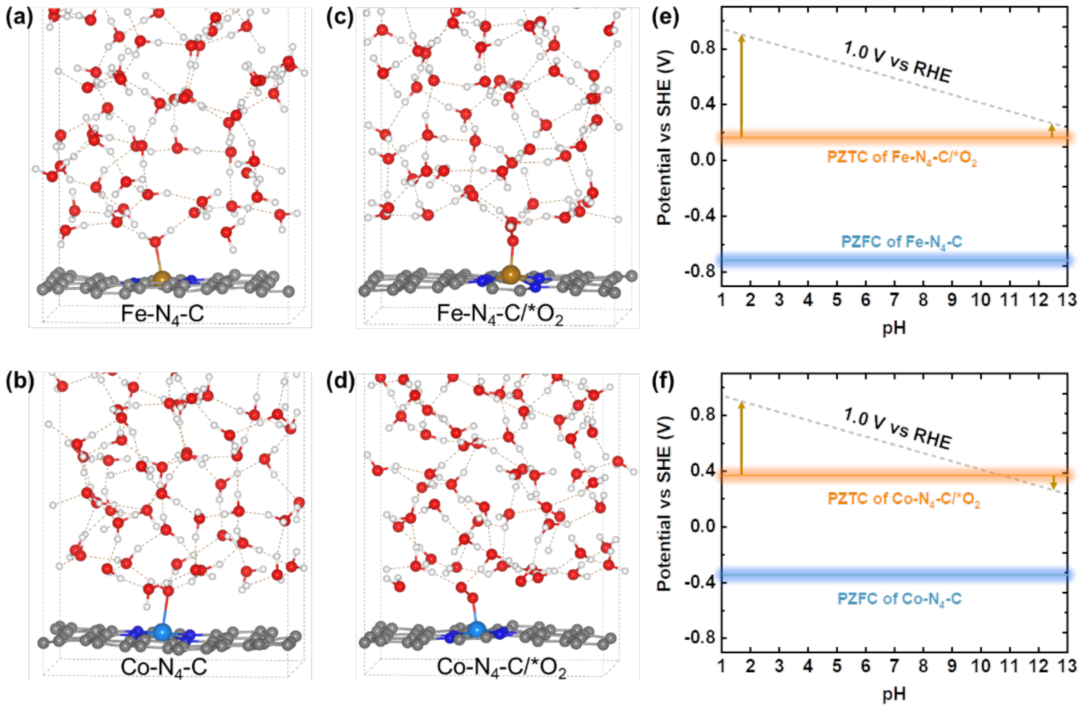

Supplementary Figure 4. (a,b) Representative snapshots of Fe-N$_4$-C/water and Co-N$_4$-C/water interfaces. (c,d) Representative snapshots of Fe-N$_4$-C/water and Co-N$_4$-C/water interfaces with *O$_2$. (e,f) Pourbaix diagram showing the pH dependence of the ORR reaction potential (1.0 V vs RHE is used here), the potential of zero free charge (PZFC) and potential of zero total charge (PZTC) for Fe-N$_4$-C/water and Co-N$_4$-C/water systems.

The PZFCs for Fe-N$_4$-C and Co-N$_4$-C electrodes (Supplementary Fig. 4a,b) are calculated as -0.71 V and -0.34 V vs SHE, which are similar to the values calculated by Chan and Liu[1,2]. The PZTCs for Fe-N$_4$-C and Co-N$_4$-C electrodes with *O$_2$ (Supplementary Fig. 4c,d) are calculated as 0.17 V and 0.37 V vs SHE.

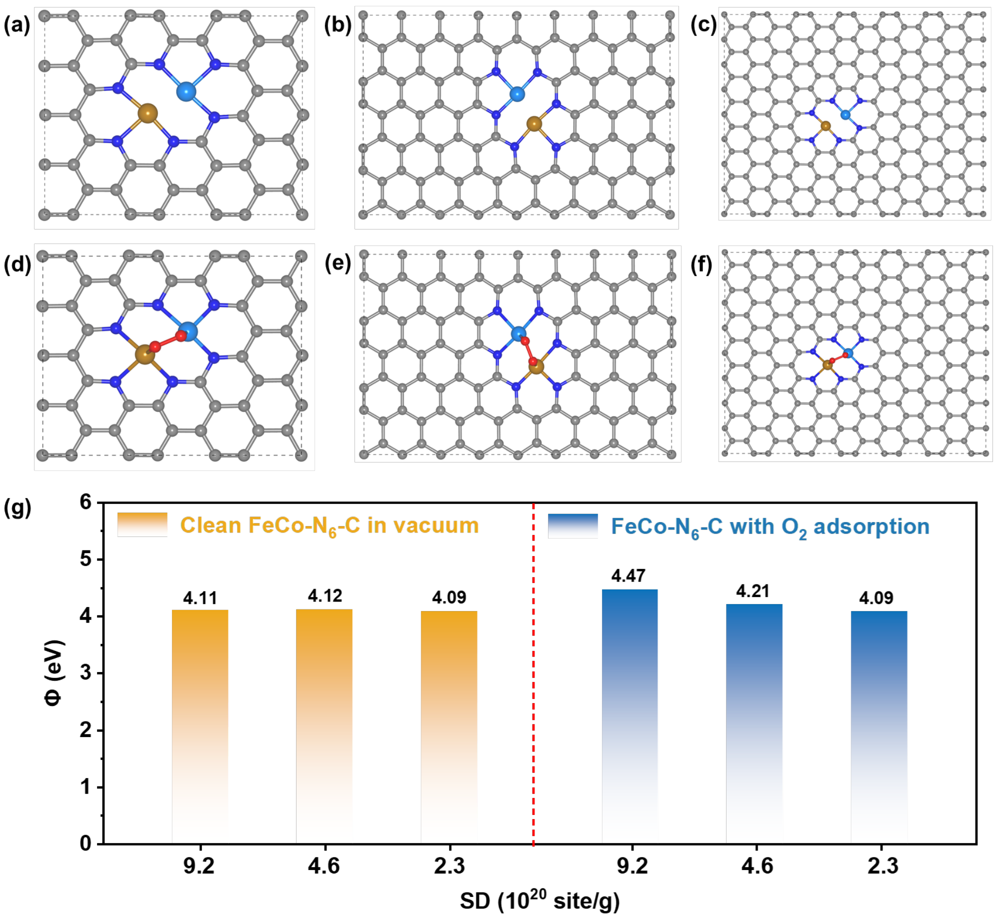

Supplementary Figure 5. (a-c) Clean and (d-f) O_2 adsorbed FeCo-N_6-C slab models with different SDs in vacuum. (a,d) 9.2×10^{20} site/g, (b,e) 4.6×10^{20} site/g and (c,f) 2.3×10^{20} site/g. (g) The calculated work functions (Φ).

Due to the limitation of computational cost when the active site density (SD) in model is similar to experimental values, the consideration of SD in the model is often ignored in most of the current ab initio simulation studies. However, evaluating the active site density in the model and its influence on the potential calculation and the results that are obtained based on the fairly small model (namely high SD) is fairly significant.

Therefore, we have estimated the SD in the synthesized FeCo-N_6-C catalyst and performed additional AIMD simulations for the interfaces with SD closer to the experimental value. Firstly, according to the composition measurement of the as-prepared FeCo-N_6-C sample by ICP-AES test[3], the experimental SD is estimated as ~1.1×10^{20} site/g, which agrees well with other reported values from 3×10^{19} site/g to 2.8×10^{20} site/g[4-7]. By contrast, the SD of the AIMD model shown in Figure 1 of the text, Supplementary Fig. 1 and Supplementary Fig. 5a,d, which is

calculated as 9.2×10^{20} site/g, is obviously higher than the experimental SD of prepared FeCo-N_6-C catalyst.

To evaluate the influence of the SD in interface model on the potential calculation and the results that are obtained based on the fairly small model (namely high SD), we have established two larger FeCo-N_6-C models (Supplementary Fig. 5b,c,e,f), which possess the SD of 4.6×10^{20} site/g and 2.3×10^{20} site/g, respectively. Such SD in the enlarged model is fairly closer to the experimental values of the as-prepared FeCo-N_6-C sample. For these three models with different SDs (Supplementary Fig. 5a-f), we have calculated the work functions (Φ) in vacuum. Supplementary Fig. 5g shows that, the Φ values hardly change for clean FeCo-N_6-C slabs while slightly decrease for O_2 adsorbed FeCo-N_6-C with the SD.

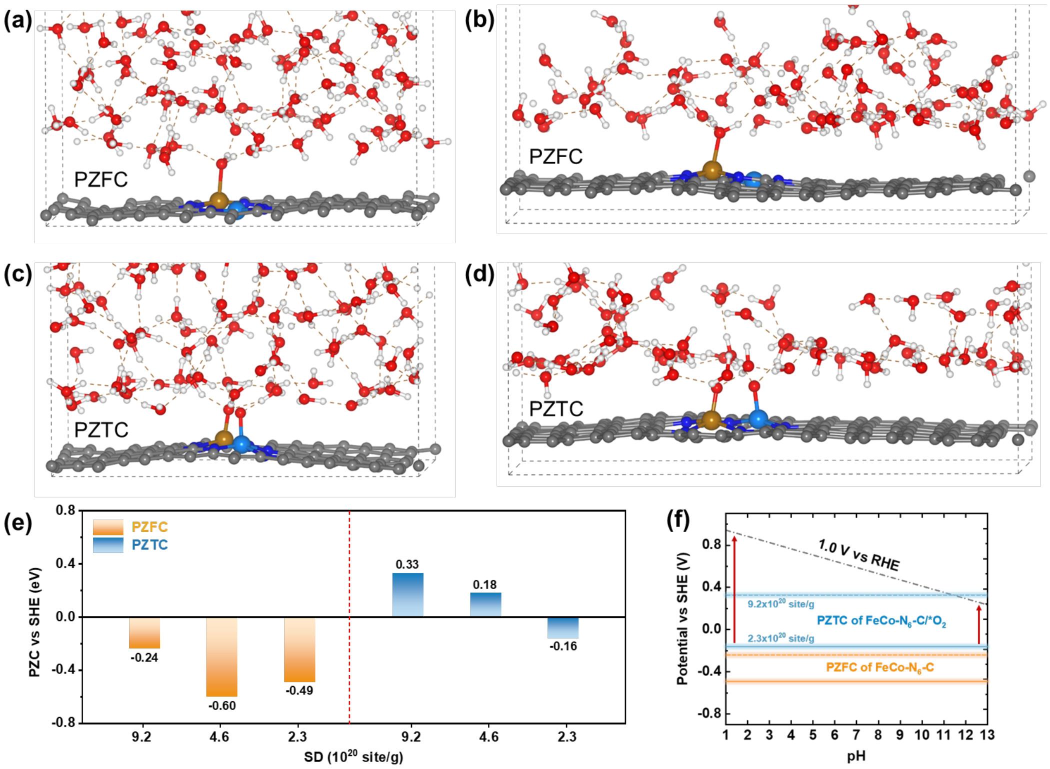

Supplementary Figure 6. (a,b) Representative snapshots of FeCo-N_6-C/water interfaces with SD of 4.6×10^{20} site/g and 2.3×10^{20} site/g, respectively. (c,d) Corresponding snapshots of FeCo-N_6-C/water interfaces with O_2 adsorption. (e) Comparison of PZC vs SHE for the clean (PZFC) and O_2 adsorbed (PZTC) FeCo-N_6-C/water interfaces with different SDs. (f) Pourbaix diagram showing the pH dependence of the ORR reaction potential (1.0 V vs RHE is used here), the PZFCs and PZTCs for FeCo-N_6-C/H_2O systems with SD of 9.2×10^{20} site/g (horizontal dashed lines) and 2.3×10^{20} site/g (horizontal solid lines).

We then performed AIMD simulations for these two enlarged FeCo-N_6-C/water interfaces without and with the O_2 adsorption (Supplementary Fig. 6a-d), and meanwhile their potentials of zero charge (PZCs) were obtained. As shown in Supplementary Fig. 6e, for the clean FeCo-N_6-C/water interfaces, the potential of zero free charge (PZFC) exhibits obvious decrease with the SD decreasing, but not monotonically. By contrast, the potential of zero total charge (PZTC) of O_2 adsorbed FeCo-N_6-C/water interface decreases monotonically with the SD decreasing. Such change trends of the PZTC and PZFC for FeCo-N_6-C/water interfaces as well as the Φ for the O_2 adsorbed FeCo-N_6-C indicate that the SD in model indeed affects the magnitude of the surface dipole potential induced by intermediate and thus the calculated value of the electrode potential.

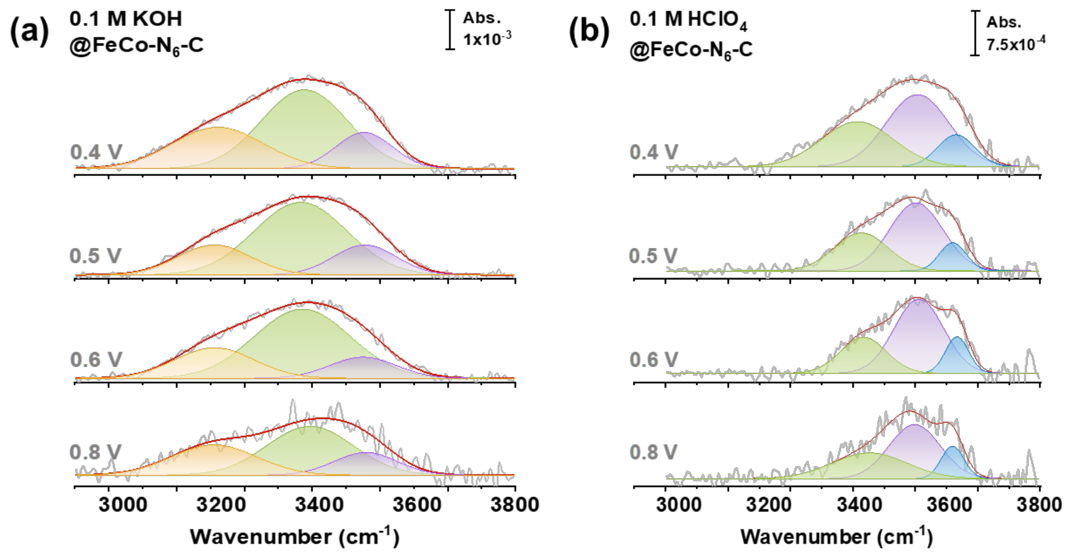

Supplementary Figure 7. Deconvolution of O-H stretching features of in situ SEIRAS spectra (grey curves) of ORR on FeCo-N_6-C recorded from 0.8 V to 0.4 V vs RHE in O_2 saturated (a) 0.1 M KOH and (b) 0.1 M $HClO_4$ solutions. Spectra were subtracted by the reference spectrum taken at 1.1 V vs RHE. The OH stretching peaks were deconvoluted into three components in both alkaline and acid systems.

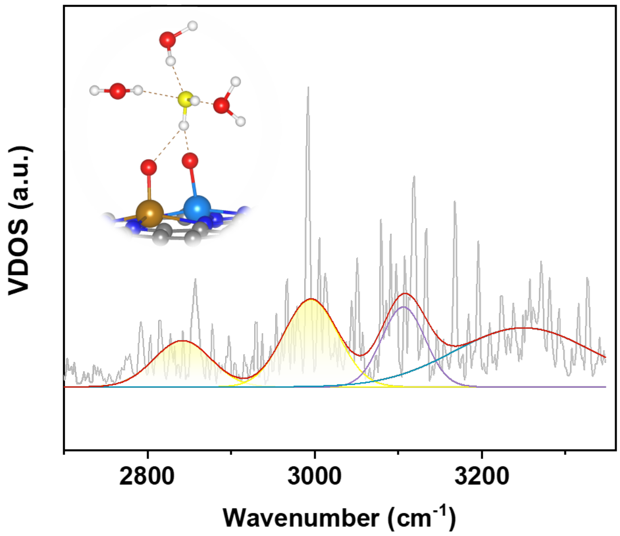

Supplementary Figure 8. The computational VDOS (grey curve) of the OH stretching feature of interfacial water molecules that form hydrogen bonds with the surface oxygenated intermediates at O_2 adsorbed alkaline interface. The Gaussian fitting has been performed.

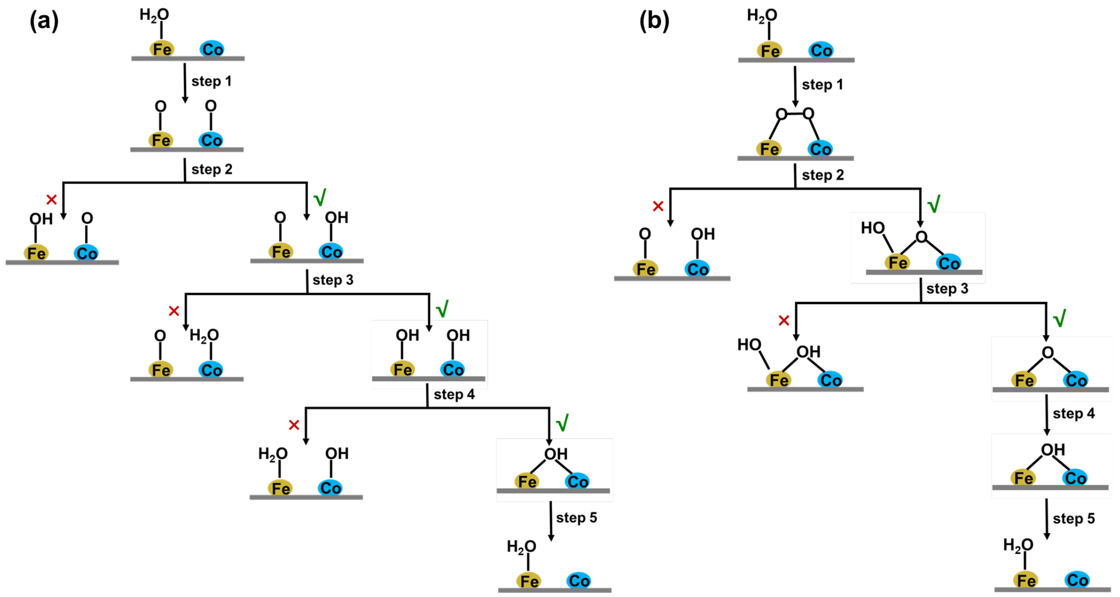

Supplementary Figure 9. Schematic diagrams of the determination processes of ORR mechanisms under (a) alkaline and (b) acid conditions. In each step, all possible reaction products have been simulated through AIMD to obtain the statistical average of total energy for comparison. The green ticks represent the selected final products, and the red crosses represent the excluded product states with higher energy.

(a)

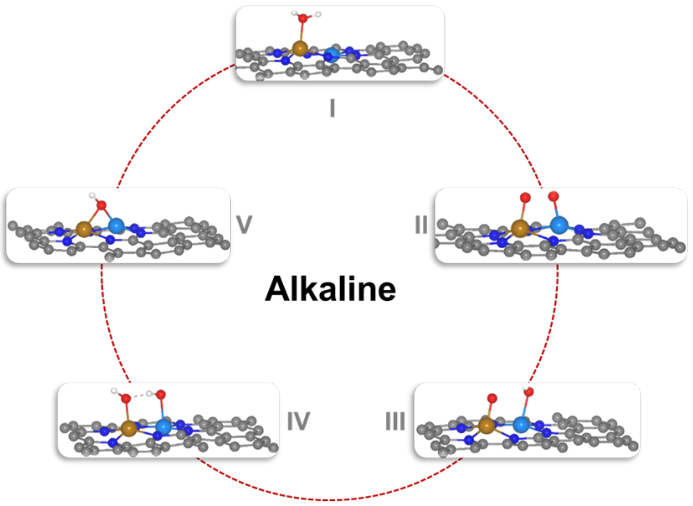

(b)

**Alkaline ORR mechanism**		
(Co)-H_2O(Fe) + O_2 → *O(Co)-*O(Fe) + H_2O	R-1	Chemisorption
*O(Co)-*O(Fe) + H_2O + e^- → *OH(Co)-*O(Fe) + OH^-	R-2	PCET
*OH(Co)-*O(Fe) + H_2O + e^- → *OH(Co)-*OH(Fe) + OH^-	R-3	PCET
*OH(Co)-*OH(Fe) + H_2O + e^- → *OH(Co-Fe) + H_2O + OH^-	R-4	PCET
*(Co)-*OH(Fe) + H2O + e^- → *(Co)-*H_2O(Fe) + OH^-	R-5	PCET

Supplementary Figure 10. (a) Close-up of the ORR process at alkaline interface. The electrolyte environment is not displayed. (b) The corresponding elementary reaction equations along the determined ORR pathway at alkaline interface. The H_2O marked by red color represent the interfacial water molecules which serve as the proton donors and provide H atoms to surface oxygenated intermediates, while the H_2O marked by green color mean the reaction products of ORR.

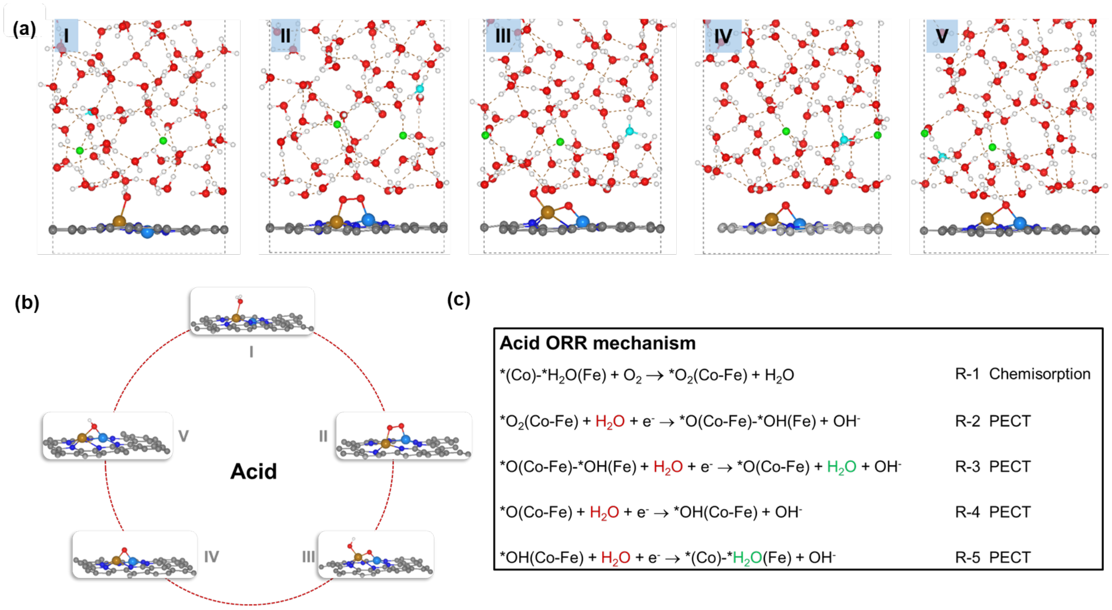

Supplementary Figure 11. (a) Representative snapshots of the interfacial structures along the ORR process in acid media. (b) Close-up of the ORR process at acid interface. The electrolyte environment is not displayed. (c) The corresponding elementary reaction equations along the determined ORR pathway at acid interface. The H_2O marked by red color represent the interfacial water molecules which serve as the proton donors and provide H atoms to surface oxygenated intermediates, while the H_2O marked by green color mean the reaction products of ORR. The generated OH^- species will be naturally neutralized by hydronium ions in bulk solution, which is not displayed here.

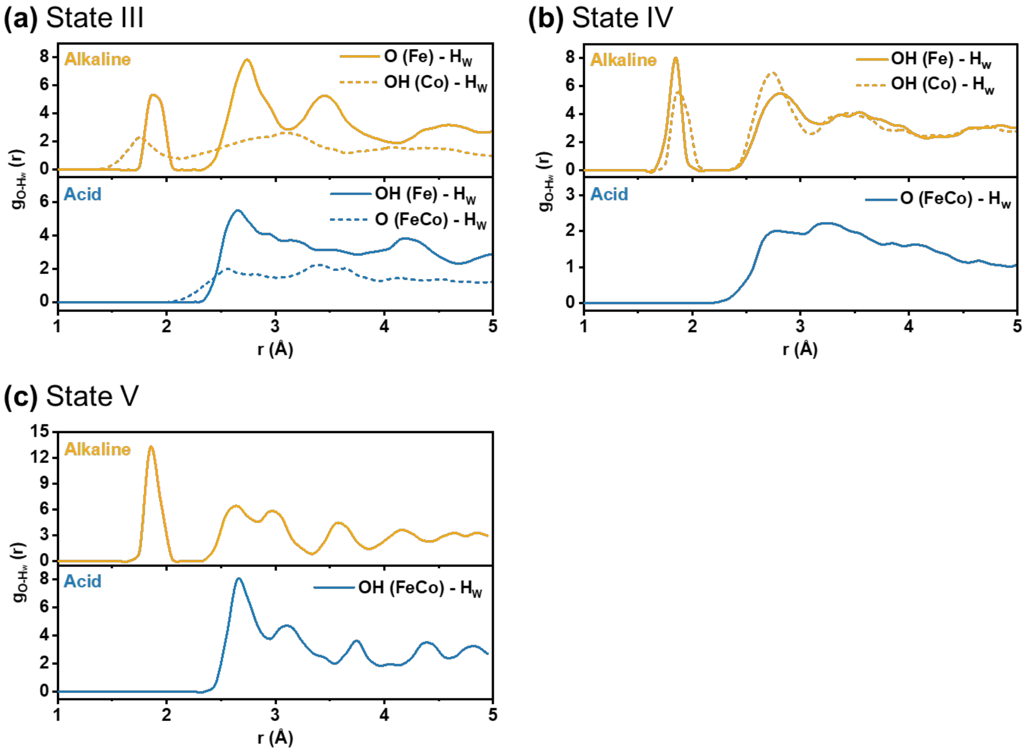

Supplementary Figure 12. Radial distribution functions between the O atoms of surface oxygenated intermediates in various reaction intermediate states and the H atoms of interfacial water at alkaline and acid interfaces. Figures a-c correspond to the state III-V, respectively, as shown in Fig. 4c and Supplementary Fig. 11a.

At each reaction intermediate state of alkaline ORR, it is apparent that the radial distribution functions exhibit sharp peaks around 1.85 Å, which indicates the formation of hydrogen bonds between the O atoms of various oxygenated intermediates with the H atoms of interfacial water in alkaline media. By contrast, the radial distribution functions for acid interface do not exhibit peaks within ~2.35 Å, indicating the missing of such hydrogen bonds.

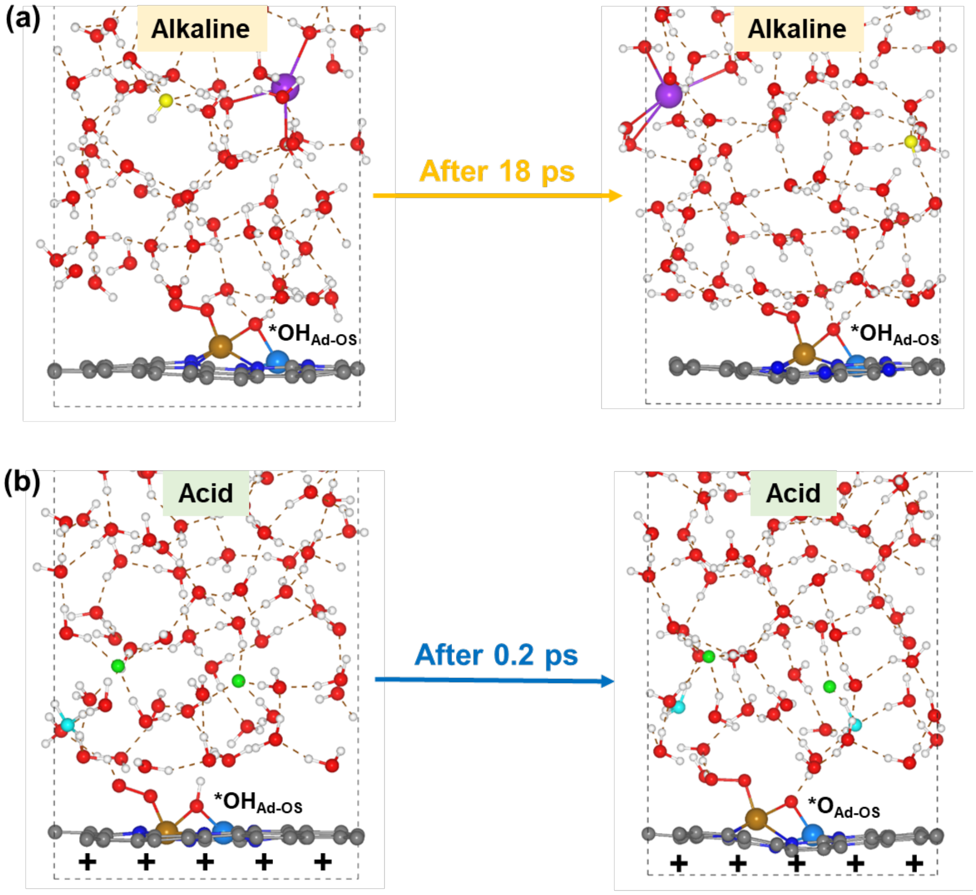

Supplementary Figure 13. The interface structures in (a) alkaline and (b) acid medias before and after AIMD simulations.

It is known that the oxygenated species, *OH or *O, often occupy the surface sites and act as the spectators in ORR process. For DAC catalysts, these strong adsorption of oxygenated intermediates on the bridge site of two metal atoms (see State V in Fig. 4a and States III-V in Fig. 4b for examples) is very likely to result in the existence of oxygenated spectators. Such scenario has been considered in this work.

To determine what exactly the surface oxygenated spectators are in alkaline and acid medias under experiment ORR potentials, the *OH oxygenated species on the Fe-Co bridge sites are evaluated, as shown in the left panels of Supplementary Fig. 13a,b. At alkaline interface, the *OH occupying the Fe-Co bridge site remains stable throughout the 18 ps AIMD simulation (Supplementary Fig. 13a), which implies that the oxygenated spectator should be *OH (termed as $*OH_S$). While at acid interface, the *OH occupying the Fe-Co bridge site is oxidized spontaneously and turns into a *O species and a H_3O^+ cation after merely 0.2 ps AIMD

simulation (Supplementary Fig. 13b), which indicates that the oxygenated spectator should be *O (termed as $*O_S$). This difference in oxygenated spectator between alkaline and acid systems can be attributed to the difference in surface charge densities of FeCo-N_6-C electrodes. Clearly, the positively charged FeCo-N_6-C electrode can induce the transition of *OH to *O.

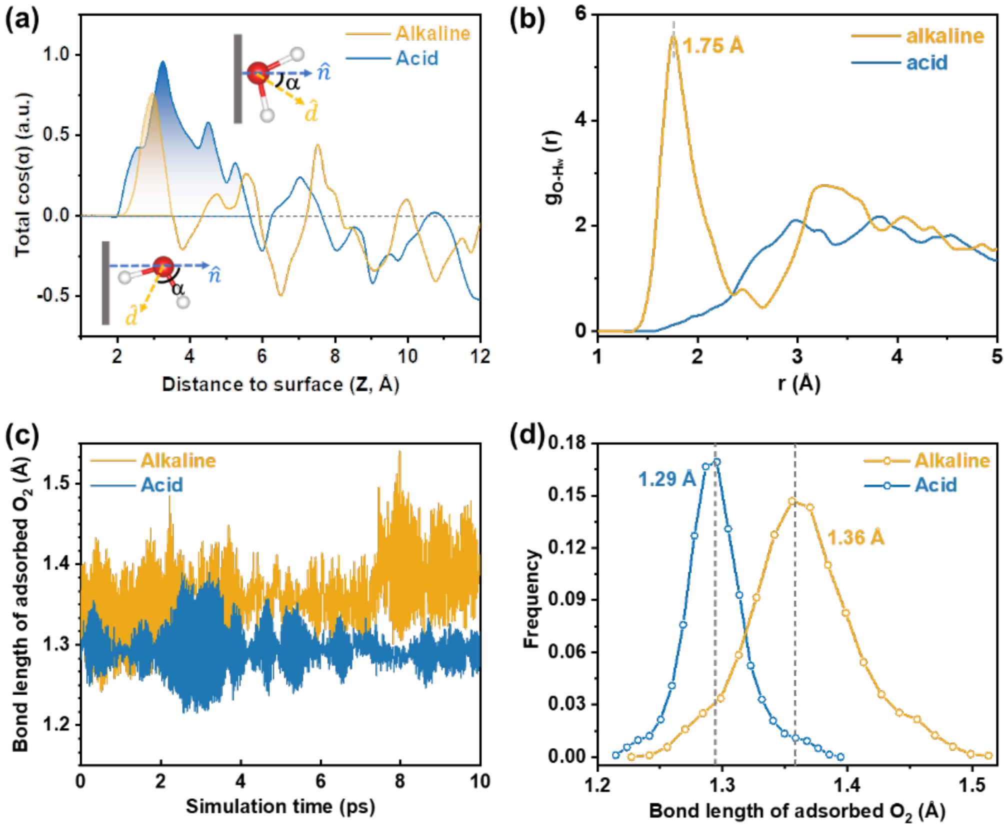

Supplementary Figure 14. (a) Distribution profiles of water dipole orientations along the surface normal direction at acid and alkaline interfaces when the $*OH_{Ad-OS}$ and $*O_{Ad-OS}$ are considered. The insets show that α is defined as the angle between the vector of water dipole ($\hat{d}$) and the surface normal ($\hat{n}$). (b) Radial distribution functions between the O atom of adsorbed O_2 molecule that points to the solution and the H atoms of interfacial water. (c) The extraction of O-O bond length of the adsorbed O_2 molecule during the 10 ps AIMD product simulations for alkaline and acid systems. (d) Statistical distributions of the O-O bond length of adsorbed O_2 molecule at acid and alkaline interfaces.

The distribution of total cosα displays a series of peaks with much higher amplitudes within ~5.6 Å at acid interface while only a fairly weak and narrow peak within ~3.5 Å at alkaline interface (Supplementary Fig. 14a). This implies that despite the presence of $*OH_{Ad-OS}$ and $*O_{Ad-OS}$, the interfacial water molecules are still orientated orderly in the form of O-down configuration due to the positively charged electrode surface in acid media, while disorderly in alkaline media. Correspondingly, the O atom of end-on adsorbed O_2 that points to the solution can form the hydrogen bonds with the H atoms of interfacial water in alkaline media (Supplementary Fig.

14b). Furthermore, it can be seen that the O-O bond length of adsorbed O_2 molecule at alkaline interface is obviously larger than that at acid interface (1.36 Å vs 1.29 Å), due to the assistance of hydrogen bonds formed with interfacial water (Supplementary Fig. 14c,d). Notably, compared with the O-O bond length (1.90 Å) at the alkaline interface without oxygenated spectators (Figs. 1a and 2d), the adsorbed O_2 molecule in the end-on configuration at the alkaline interface with the existence of $*OH_{Ad\text{-}OS}$ does not dissociate.

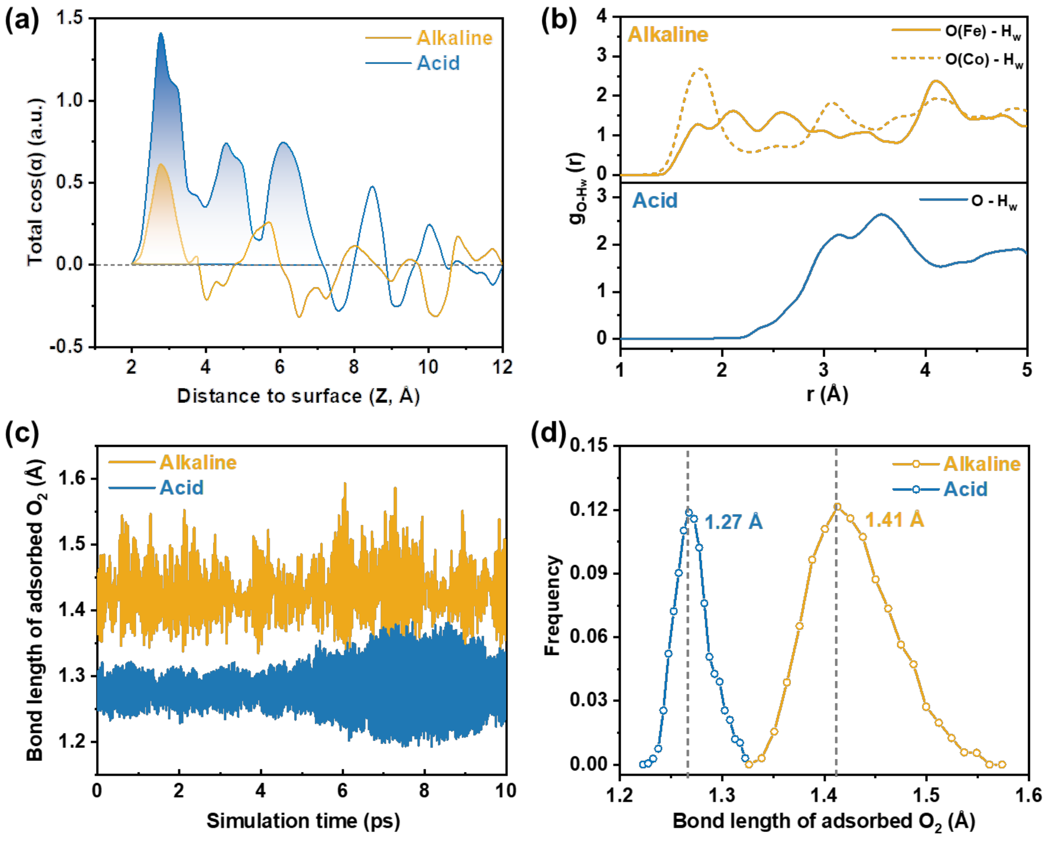

Supplementary Figure 15. (a) Distribution profiles of water dipole orientations along the surface normal direction at acid and alkaline interfaces when the $*OH_{Ax\text{-}OS}$ and $*O_{Ax\text{-}OS}$ are considered. The insets show that α is defined as the angle between the vector of water dipole ($\hat{d}$) and the surface normal ($\hat{n}$). (b) Radial distribution functions between the O atom of adsorbed O_2 molecule and the H atoms of interfacial water. (c) The extraction of O-O bond length of the adsorbed O_2 molecule during the 10 ps AIMD product simulations for alkaline and acid systems. (d) Statistical distributions of the O-O bond length of adsorbed O_2 molecule at acid and alkaline interfaces.

The distribution of total cosα displays a series of peaks with much higher amplitudes within ~7.1 Å at acid interface while only a fairly weak and narrow peak within ~3.5 Å at alkaline interface (Supplementary Fig. 15a). This implies that despite the presence of $*OH_{Ax\text{-}OS}$ and $*O_{Ax\text{-}OS}$, the interfacial water molecules are still orientated orderly in the form of O-down configuration due to the positively charged electrode surface in acid media, while disorderly in alkaline media. Correspondingly, the O atoms of adsorbed O_2 can form hydrogen bonds with the H atoms of interfacial water in alkaline media (Supplementary Fig. 15b). Furthermore, as depicted in

Supplementary Fig. 15c,d, it can be seen that although the adsorption configurations of O_2 molecule at alkaline and acid interfaces are distinct, the O-O bond length of adsorbed O_2 molecule at alkaline interface is still obviously larger than that at acid interface (1.41 Å vs 1.27 Å), due to the assistance of hydrogen bonds formed with interfacial water.

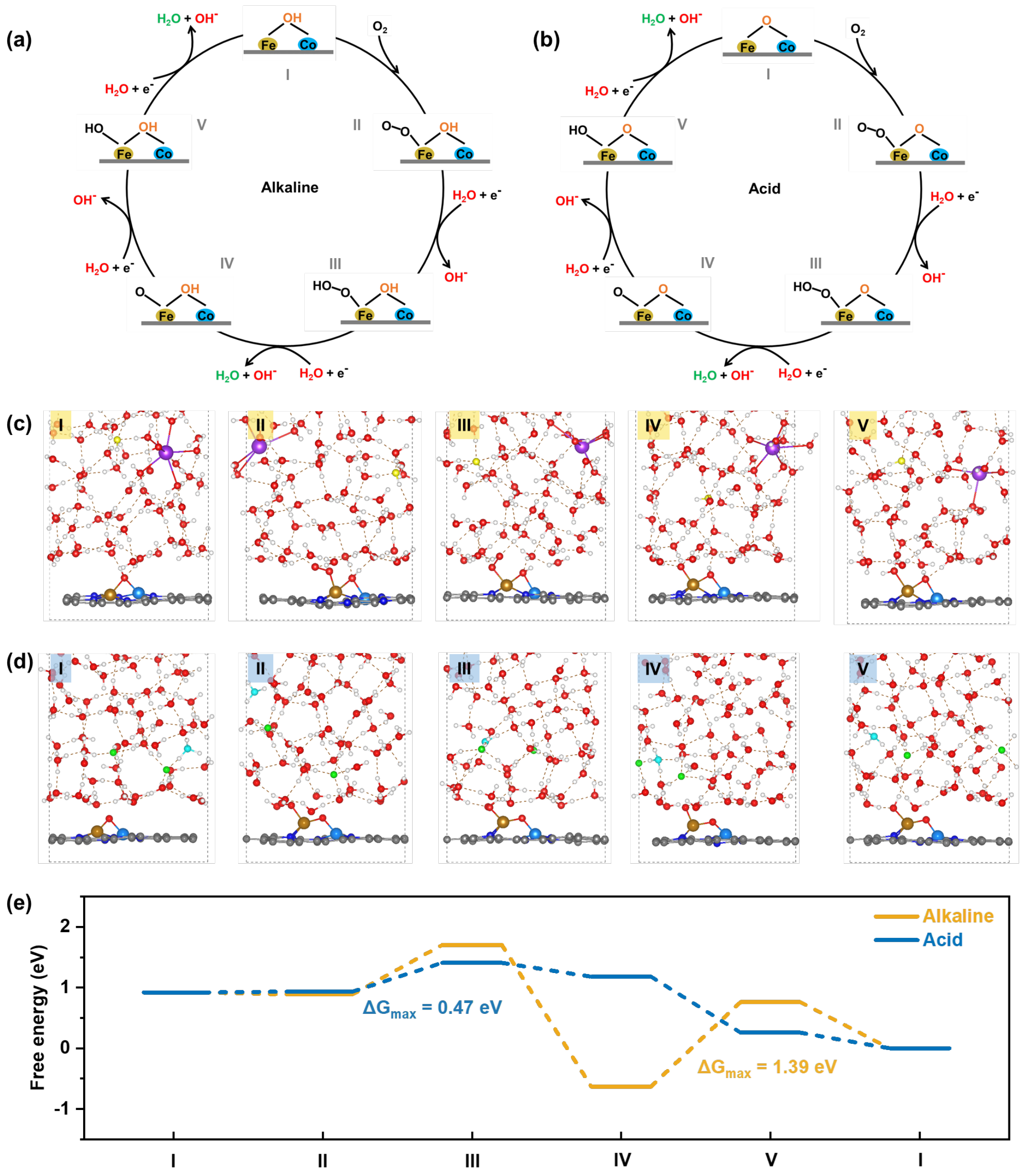

Supplementary Figure 16. (a,b) Schematic diagrams of ORR processes in (a) alkaline and (b) acid medias when the $*OH_{Ad\text{-}OS}$ and $*O_{Ad\text{-}OS}$ (marked by orange color) are considered. The H_2O marked by red color represent the water molecules which locate in the solution and provide proton to surface oxygenated intermediates, while the H_2O marked by green color mean the reaction products. (c,d) Representative snapshots of the interfacial structures along the ORR processes in (c) alkaline and (d) acid medias. (e) Free energy diagrams for ORR at alkaline and acid interfaces for U = 1.0 V vs RHE.

The determined ORR pathways at alkaline and acid interface when the Ad-OS are considered are show in Supplementary Fig. 16a,b, and the corresponding interface structures of various

reaction intermediate states are shown in Supplementary Fig. 16c,d. It can be noted that due to the existence of $*OH_{Ad\text{-}OS}$ and $*O_{Ad\text{-}OS}$, the Co atom with relatively weak oxygenophilicity has been poisoned and thereby the synergistic effect between adjacent metal active sites is broken. Therefore, at both alkaline and acid interfaces with the existence of Ad-OS, the adsorbed O_2 molecules in the end-on configurations do not dissociate (Fig. 5a,b in the text and Supplemental Fig.16), resulting in the formation of the *OOH intermediates. At this time, it can be realized that the FeCo-N_6-C double atom catalyst is similar to the Fe-N_3O-C single atom catalyst to a certain extent.

Furthermore, Supplementary Fig. 16e shows that in the presence of $*OH_{Ad\text{-}OS}$ and $*O_{Ad\text{-}OS}$, the third PCET step (IV→V) is the PDS of alkaline ORR; while for acid ORR, the PDS is the first PCET step (II→III). Notably, the free energy change of PDS at acid interface is still much smaller than that at alkaline interface (0.47 eV vs 1.39 eV), contradicting the experimental truth that the ORR activity in alkaline is superior to that in acid.

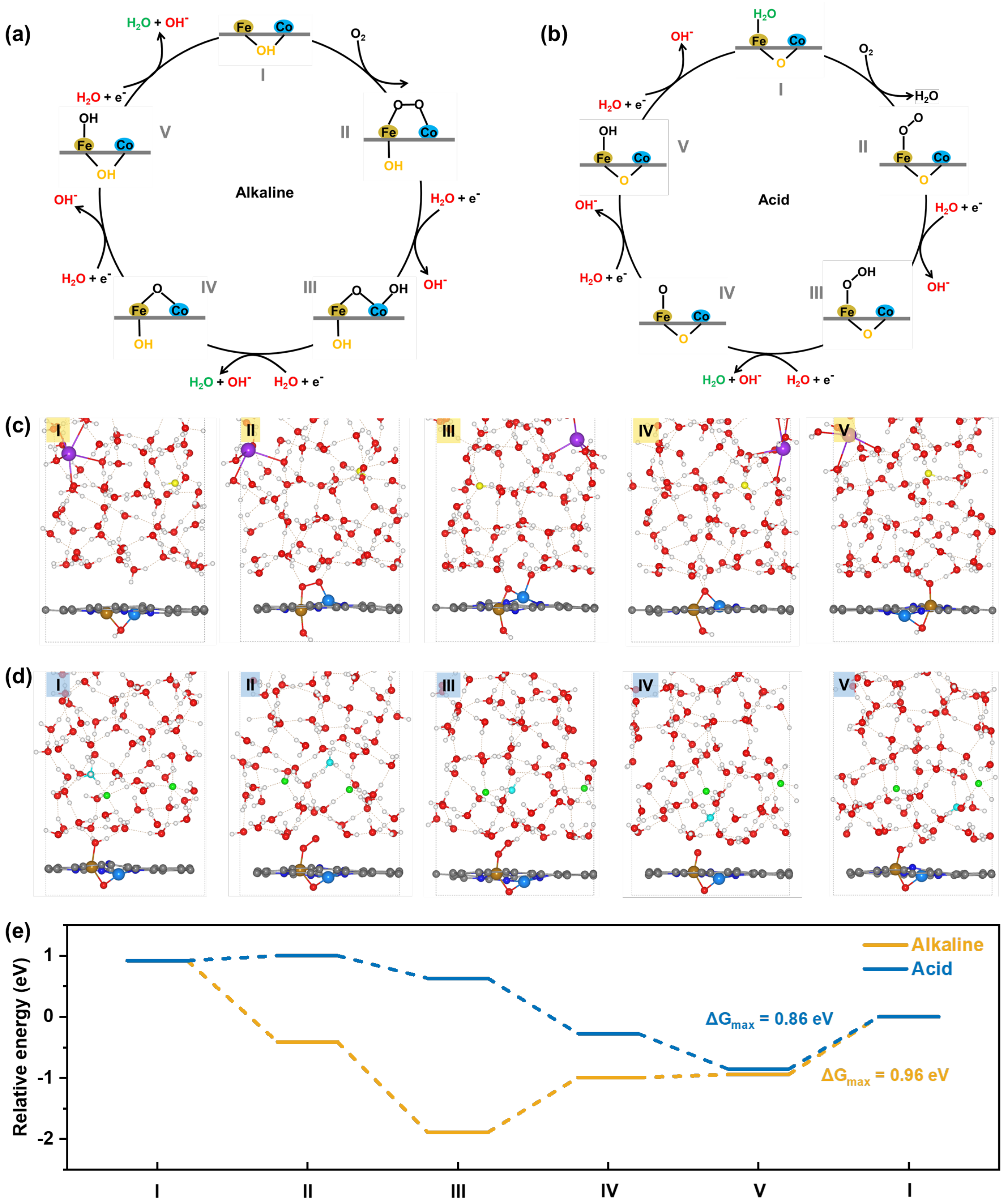

Supplementary Figure 17. (a,b) Schematic diagrams of ORR processes in (a) alkaline and (b) acid medias when the $^{*}OH_{Ax\text{-}OS}$ and $^{*}O_{Ax\text{-}OS}$ (marked by yellow color) are considered. The H_2O marked by red color represent the water molecules which locate in the solution and provide proton to surface oxygenated intermediates, while the H_2O marked by green color mean the reaction products. (c,d) Representative snapshots of the interfacial structures along the ORR processes in (c) alkaline and (d) acid medias. (e) Free energy diagrams for ORR at alkaline and acid interfaces for U = 1.0 V vs RHE.

The determined ORR pathways at alkaline and acid interface when the Ax-OS are considered

are show in Supplementary Fig. 17a,b, and the corresponding interface structures of various reaction intermediate states are shown in Supplementary Fig. 17c,d. It can be noted that the O_2 molecule adsorbs on the Fe-Co bridge site at alkaline interface with the existence of $*OH_{Ax-OS}$, thus leading to the dissociative mechanism of ORR. While, due to the strong interaction between $*O_{Ax-OS}$ and Fe-Co bridge site, the interaction between Co atom with relatively weak oxygenophilicity and O atom of adsorbed O_2 has been greatly weakened, thus resulting in that the Co atom cannot adsorb O_2 to form the Fe-Co bridge adsorption configuration. In other words, at acid interfaces with the existence of $*O_{Ax-OS}$, the adsorbed O_2 molecule is in the end-on configuration, resulting in the associative mechanism of ORR.

Furthermore, Supplementary Fig. 17e shows that in the presence of $*OH_{Ax-OS}$ and $*O_{Ax-OS}$, the fourth PCET steps (V→I) are the PDS of both alkaline and acid ORR. Notably, the free energy change of PDS at acid interface is still smaller than that at alkaline interface (0.86 eV vs 0.96 eV), contradicting the experimental truth that the ORR activity in alkaline is superior to that in acid.

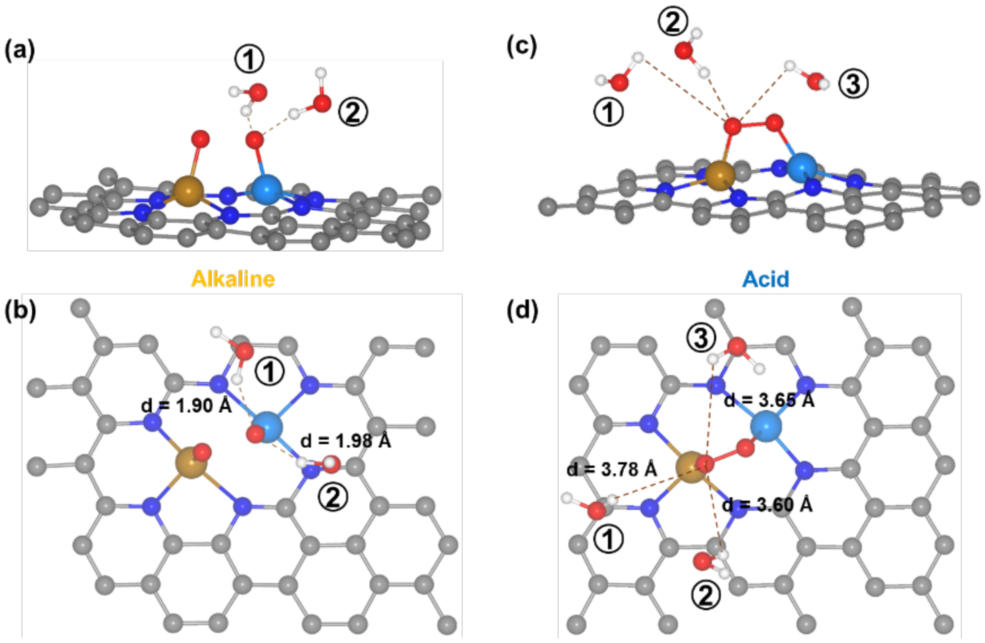

Supplementary Figure 18. (a,b) Close-up of two interfacial water molecules that form hydrogen bonds (brown dashed lines) with the O atom on Co site for alkaline system. (a) side view; (b) top view. The indexes of these two water molecules and the corresponding length of hydrogen bonds are shown. (c,d) Close-up of three interfacial water molecules closest to the O atom on Fe site for acid system. (c) side view; (d) top view. The indexes of these three water molecules and the corresponding distances between the H atom of water and O atom on Fe site are shown.

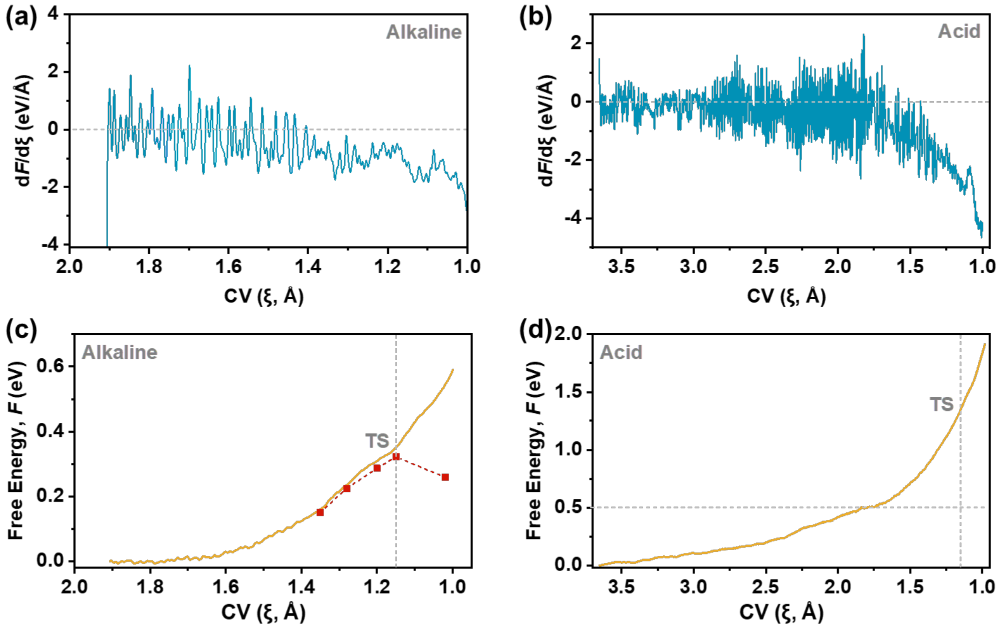

Supplementary Figure 19. (a,b) Potential of mean force profiles derived from the slow-growth simulations for the first PCET reaction at alkaline and acid interfaces, respectively. (c,d) The free energy profiles (yellow solid curves) along the collective variable (CV, ξ) at alkaline and acid interfaces, respectively. The red dashed curve in (c) means the free energy profile after constant potential correction.

Supplementary Figure 19 shows the typical potential of mean force profiles derived from the slow-growth simulations for the first PCET reactions at alkaline and acid interfaces and the corresponding free energy profiles along the collective variable (CV, ξ). It can be seen that the free energy profiles present a gradual upward trend rather than a parabola-like shape[1,8]. It may be due to the fact that, the slow-growth simulation was performed under constant charge condition, and thus the charge density on FeCo-N_6-C electrode varies greatly, when interfacial water molecule donates a hydrogen atom to the oxygen-containing reaction intermediate and generates a OH^- anion, especially around the transition state (TS). As shown in Supplementary Fig. 19b, after the constant potential correction as described in Supplementary Note 1, the typical parabolic free energy profile (red dashed curve) of an elementary reaction process can be well obtained, and the CV of TS is determined as ~1.15 Å. Such value has also been used to determine the TS in the slow-growth simulation at acid interface (Supplementary Fig. 19d). Note that even within a fairly large CV range around 1.15 Å at acid interface, the intermediate

state is always much higher in free energy than that of TS for alkaline interface, which unequivocally demonstrates that the PECT reaction at acid interface is very sluggish.

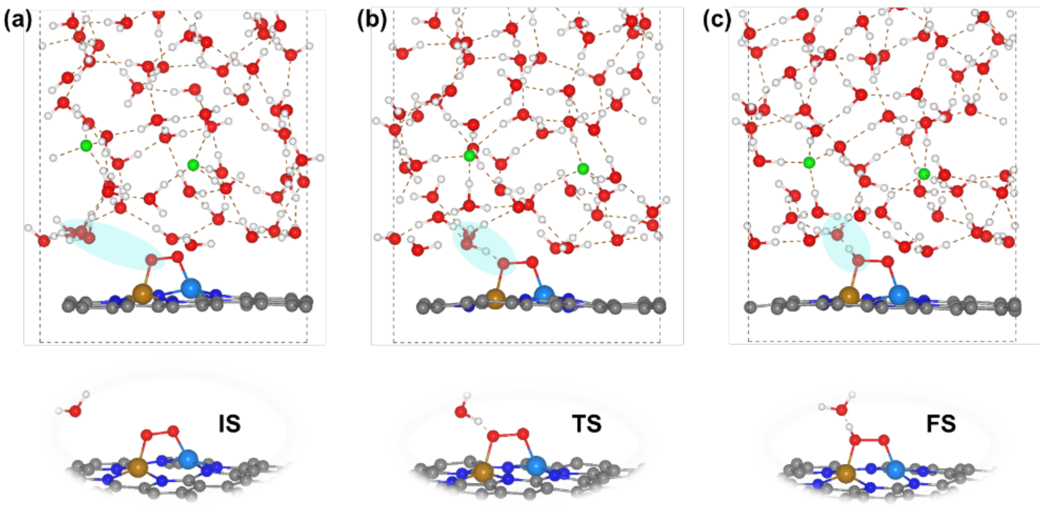

Supplementary Figure 20. (a-c) The interface structures (upper panel) and corresponding close-up (lower panel) of the initial state (IS), transition state (TS) and final state (FS) in one typical slow-growth simulation for the first PCET reaction of acid ORR.

At acid interface, in the process from IS to TS, the reactive water molecule first gradually approaches the O atom on Fe site with maintaining the O-down configuration, and then its orientation is flipped to the 'one-H-down' configuration in order to provide the H atom to the O atom of adsorbed O_2 molecule. Due to the strong electrostatic interaction to interfacial water molecule exerted by the positively charged FeCo-N_6-C electrode, the flip of water dipole orientation contributes to a fairly high barrier. The following process from TS to FS corresponds to the bond formation between the O atom of O_2 on Fe site and the H atom of reactive water molecule, and meanwhile the generated OH^- anion turns into water molecule by receiving a H atom from another water molecule that locates further away from the electrode.

Supplementary Table 1. Average free energy barriers ($\Delta G^{\neq}$) and error bars for various slow-growth simulations of the first PCET reactions at alkaline and acid interfaces.

Simulation ID	$\Delta G^{\neq}$ (eV)	Error bar (eV)
Alkaline-1	0.328	0.022
Alkaline-2	0.286	0.027
Acid-1	1.318	0.014
Acid-2	1.344	0.019
Acid-3	2.149	0.059

Supplementary Note 1: constant potential correction.

In the actual electrocatalytic reaction process, the electrode potential always keeps constant. However, in the AIMD simulation, it is the charge of the system that keeps constant; while the work function and electrode potential changes moment by moment along the reaction process. Thus, we need to correct the calculated energy change to conform the constant-potential condition.

For a constant-charge simulation from state 1 to state 2, we can calculate the corresponding total energies $E_1(\Phi_1)$ and $E_2(\Phi_2)$, work functions Φ_1 and Φ_2, as well as the number of excess electrons in the slabs q_1 and q_2 using Bader analysis. According to the works of Chan and Nørskov[9,10], we can use these data to calculate the corresponding energy change between the two states at a certain work function. For example, we can calculate the energy change at constant potential Φ_1 using the following equation:

$$E_2(\Phi_1) - E_1(\Phi_1) = E_2(\Phi_2) - E_1(\Phi_1) + \frac{(q_2 - q_1)(\Phi_2 - \Phi_1)}{2} \quad (1)$$

Supplementary Note 2: slow-growth approach

The free energy profile along a collective variable (CV, ξ) can be scanned by an approximate slow-growth approach[11]. In this method, the value of ξ is linearly changed from the value characteristic for state 1 to that for state 2 with a velocity of transformation $\dot{\xi}$. The resulting work needed to perform a transformation 1→2 can be computed as:

$$w_{1\to2}^{irrev} = \int_{\xi_1}^{\xi_2} (\frac{\partial F(q)}{\partial \xi}) \cdot \dot{\xi} dt \qquad (2)$$

where $F(q)$ is the free energy at general coordinate q which is evolving with time t, $\frac{\partial F}{\partial \xi}$ is calculated along the track of a constrained MD through the SHAKE algorithm.

In the limit of infinitesimally small $\dot{\xi}$, the work $w_{1\to2}^{irrev}$ corresponds to the free-energy difference between state 1 and state 2. In the general case, $w_{1\to2}^{irrev}$ is the irreversible work related to the free energy via Jarzynski's identity:

$$exp\left\{-\frac{\Delta F_{1\to2}}{k_B T}\right\} = \langle exp\left\{-\frac{w_{1\to2}^{irrev}}{k_B T}\right\}\rangle \qquad (3)$$

where $\langle ... \rangle$ stand for the statistical average of the term enclosed in angular parentheses.

Supplementary Note 3: The free energy diagram calculation

For the chemisorption step of O_2 molecule, the free energy change is calculated by the following equation:

$$\Delta G = \left(\langle E_{*O_2}\rangle + ZPE_{*O_2} - TS_{*O_2}\right) - \langle E_*\rangle - \left(E_{O_2} + ZPE_{O_2} - TS_{O_2}\right) - \Delta E_{corr} \qquad (4)$$

where $\langle E_{*O_2}\rangle$ and $\langle E_*\rangle$ are the statistically averaged internal energies of interface systems before and after O_2 adsorption; ZPE and TS are the corresponding zero-point energy and vibrational entropy of reaction intermediate; E_{H_2} is the internal energy of H_2(g).

For each PCET reaction in the alkaline and acid ORR, the free energy change is calculated by the following equation:

$$\Delta G = (\langle E_{*B}\rangle + ZPE_{*B} - TS_{*B}) - (\langle E_{*A}\rangle + ZPE_{*A} - TS_{*A}) - \frac{1}{2}\left(E_{H_2} + ZPE_{H_2} - TS_{H_2}\right) + eU_{cal}$$
$$+e(U - U_{cal}) + \Delta E_{corr} \qquad (5)$$

where $\langle E_{*A}\rangle$ and $\langle E_{*B}\rangle$ are the statistically averaged internal energies of interface systems before and after PCET reaction (A and B represent the surface reaction intermediates); ZPE and TS are the corresponding zero-point energy and vibrational entropy of reaction intermediate; E_{H_2} is the internal energy of H_2(g). If the PCET reaction generates water molecule product, the ΔG should also add the term $E_{H_2O} + ZPE_{H_2O} - TS_{H_2O}$, in which the E_{H_2O} is the energy of liquid water molecule in AIMD simulation, rather than that of an individual water molecule in vacuum. All values of *ZPE* and *TS* are from previous reports[12,13]. Because the work function changes after reaction, the constant potential correction has been performed, and the correction term ΔE_{corr} equals to $\frac{(q_2-q_1)(\Phi_2-\Phi_1)}{2}$, in which $\Phi_2 - \Phi_1$ and $q_2 - q_1$ are the changes in work function and charge of the FeCo-N_6-C electrode plus all adsorbates, respectively, from state 1 to state 2. The '+' and '-' symbols represent that the corrections are performed at constant potential Φ_2 and Φ_1, respectively, which is closer to the work function of *O_2 reaction intermediate state (viz. the state I of ORR process) is chosen as the constant potential. In addition, in the construction of the free energy diagrams for ORR, the electrode potentials (U) for alkaline and acid systems have been set as the same value (1.0 V vs RHE), which is convenient for the comparison of the reaction thermodynamics. Therefore, the ΔG of an PCET step has been adjusted by the term $e(U - U_{cal})$, in which the U_{cal} is the calculated electrode potential of the AIMD simulated interfaces shown in Fig. 1 and Fig. 5a-d in the text.

In other words, the second correction term $e(U - U_{cal})$ is performed, in which U is the target potential (1.0 V vs RHE) and U_{cal} is the actual potential of AIMD simulated acid or alkaline system (viz. 0.88 V or 1.10 V vs RHE), so as to compare the reaction free energy diagrams in alkaline and acid at the same electrode potential.

Supplementary References

1. Zhao, X. & Liu, Y. Origin of selective production of hydrogen peroxide by electrochemical oxygen reduction. *J. Am. Chem. Soc.* **143**, 9423-9428 (2021).

2. Vijay, S., Gauthier, J. A., Heenen, H. H., Bukas, V. J., Kristoffersen, H. H. & Chan, K. Dipole-field interactions determine the CO_2 reduction activity of 2D Fe-N-C single-atom catalysts. *ACS Catal.* **10**, 7826-7835 (2020).

3. Wang, J., Huang, Z., Liu, W., Chang, C., Tang, H., Li, Z., Chen, W., Jia, C., Yao, T., Wei, S., Wu, Y. & Li, Y. Design of N-coordinated dual-metal sites: a stable and active Pt-free catalyst for acidic oxygen reduction reaction. *J. Am. Chem. Soc.* **139**, 17281-17284 (2017).

4. Mehmood, A., Gong, M., Jaouen, F., Roy, A., Zitolo, A., Khan, A., Sougrati, M., Primbs, M., Bonastre, A., Fongalland, D., Drazic, G., Strasser, P. & Kucernak, A. High loading of single atomic iron sites in Fe-NC oxygen reduction catalysts for proton exchange membrane fuel cells. *Nat. Catal.* **5**, 311-323 (2022).

5. Luo, F., Choi, C. H., Primbs, M. J., Ju, W., Li, S., Leonard, N. D., Thomas, A., Jaouen F. & Strasser, P. Accurate evaluation of active-site density (SD) and turnover frequency (TOF) of PGM-free metal–nitrogen-doped carbon (MNC) electrocatalysts using CO cryo adsorption. *ACS Catal.* **9**, 4841-4852 (2019).

6. Leonard, N. D., Wagner, S., Luo, F., Steinberg, J., Ju, W., Weidler, N., Wang, H., Kramm U. & Strasser, P. Deconvolution of utilization, site density, and turnover frequency of Fe–nitrogen–carbon oxygen reduction reaction catalysts prepared with secondary N-precursors. *ACS Catal.* **8**, 1640-1647 (2018).

7. Sahraie, N. R., Kramm, U. I., Steinberg, J., Zhang, Y., Thomas, A., Reier, T., Paraknowitsch, J. & Strasser, P. Quantifying the density and utilization of active sites in non-precious metal oxygen electroreduction catalysts. *Nat. Commun.* **6**, 8618 (2015).

8. Li, P., Liu, Y. W. & Chen, S. L. Microscopic EDL structures and charge-potential relation on stepped platinum surface: Insights from the ab initio molecular dynamics simulations. *J. Chem. Phys.* **156**, 104701 (2022).

9. Chan, K. & Nørskov, J. K. Electrochemical barriers made simple. *J. Phys. Chem. Lett.* **6,** 2663-2668 (2015).

10. Chan, K. & Nørskov, J. K. Potential dependence of electrochemical barriers from ab initio calculations. *J. Phys. Chem. Lett.* **7,** 1686-1690 (2016).

11. Woo, T. K., Margl, P. M., Blöchl, P. E. & Ziegler, T. A combined Car−Parrinello QM/MM implementation for ab initio molecular dynamics simulations of extended systems: application to transition metal catalysis. *J. Phys. Chem. B* **101,** 7877-7880 (1997).

12. Nørskov, J. K. et al. Origin of the overpotential for oxygen reduction at a fuel-cell cathode. *J. Phys. Chem. B* **108,** 17886-17892 (2004).

13. Rossmeisl, J., Logadottir, A. & Nørskov, J. K. Electrolysis of water on (oxidized) metal surfaces. *Chem. Phys*. **319,** 178-184 (2005).
