# Supplementary material for: Revealing the role of double-layer microenvironments in pH-dependent oxygen reduction activity over metal-nitrogen-carbon catalysts
Source: Nat Commun. 2023 Oct 31;14:6936. doi: 10.1038/s41467-023-42749-7 (PMC10618200; doi:10.1038/s41467-023-42749-7)
Supplement: Supplementary file 2 — Description of Additional Supplementary Files [file 41467_2023_42749_MOESM2_ESM.docx]

**Description of Additional Supplementary Files**

**Supplementary Data 1:** Atomic coordinates of the initial and final configurations in the AIMD trajectories of O2-adsorbed alkaline and acid interfaces (in Vienna ab initio simulation package CONTCAR format).
